# Association of Interacting Genes in the Toll-Like Receptor Signaling Pathway and the Antibody Response to Pertussis Vaccination

**DOI:** 10.1371/journal.pone.0003665

**Published:** 2008-11-06

**Authors:** Tjeerd G. Kimman, Sander Banus, Naomi Reijmerink, Johan Reimerink, Foekje F. Stelma, Gerard H. Koppelman, Carel Thijs, Dirkje S. Postma, Marjan Kerkhof

**Affiliations:** 1 Center for Infectious Disease Control, National Institute of Public Health and the Environment, Bilthoven, The Netherlands; 2 Central Veterinary Institute, Lelystad, The Netherlands; 3 Department of Pulmonology, University Medical Center Groningen, University of Groningen, Groningen, The Netherlands; 4 Department of Medical Microbiology, Academic Hospital Maastricht, Maastricht, The Netherlands; 5 Department of Pediatric Pulmonology and Pediatric Allergology, Beatrix Children's Hospital, University Medical Center Groningen, University of Groningen, Groningen, The Netherlands; 6 Department of Epidemiology, School of Public Health and Primary Care (Caphri), and Nutrition and Toxicology Research Institute Maastricht (Nutrim), Maastricht University, Maastricht, The Netherlands; 7 Department of Epidemiology, University Medical Center Groningen, University of Groningen, Groningen, The Netherlands; Federal University of São Paulo, Brazil

## Abstract

**Background:**

Activation of the Toll-like receptor (TLR) signaling pathway through *TLR4* may be important in the induction of protective immunity against *Bordetella pertussis* with TLR4-mediated activation of dendritic and B cells, induction of cytokine expression, and reversal of tolerance as crucial steps. We examined whether single nucleotide polymorphisms (SNPs) in genes of the TLR4 pathway and their interaction are associated with the response to whole-cell vaccine (WCV) pertussis vaccination in 490 one-year-old children.

**Methodology/Principal Findings:**

We analyzed associations of 75 haplotype-tagging SNPs in genes in the TLR4 signaling pathway with pertussis toxin (PT)-IgG titers. We found significant associations between the PT-IgG titer and SNPs in *CD14*, *TLR4*, *TOLLIP*, *TIRAP*, *IRAK3*, *IRAK4*, *TICAM1*, and *TNFRSF4* in one or more of the analyses. The strongest evidence for association was found for two SNPs (rs5744034 and rs5743894) in *TOLLIP* that were almost completely in linkage disequilibrium, provided statistically significant associations in all tests with the lowest *p*-values, and displayed a dominant mode of inheritance. However, none of these single gene associations would withstand correction for multiple testing. In addition, Multifactor Dimensionality Reduction Analysis, an approach that does not need correction for multiple testing, showed significant and strong two and three locus interactions between SNPs in TOLLIP (rs4963060), TLR4 (rs6478317) and IRAK1 (rs1059703).

**Conclusions/Significance:**

We have identified significant interactions between genes in the TLR pathway in the induction of vaccine-induced immunity. These interactions underline that these genes are functionally related and together form a true biological relationship in a protein-protein interaction network. Practically all our findings may be explained by genetic variation in directly or indirectly interacting proteins at the extra- and intracytoplasmic sites of the cell membrane of antigen-presenting cells, B cells, or both. Fine tuning of interacting proteins in the TLR pathway appears important for the induction of an optimal vaccine response.

## Introduction

Whooping cough or pertussis is caused by the gram-negative bacterium *Bordetella pertussis*. Vaccination with both whole-cell (WCV) and acellular vaccine (ACV) limits the occurrence and severity of pertussis, but is unable to completely prevent infection and disease in vaccinated populations. Indeed, despite widespread vaccination *B. pertussis* remains endemic and has even re-emerged in many populations [1], [2]. Previous studies provided evidence for the role of the gene coding for Toll-like receptor 4 (*TLR4*) in both the infection process (in mice), and the response to vaccination (in mice and men) [3]–[10].

TLR4 was the first identified human Toll-like receptor that belongs to a class of pathogen-associated molecular pattern receptors on antigen-presenting cells, such as macrophages and dendritic cells [11]. TLR4 is the receptor for bacterial lipopolysaccharide (LPS), and is also one of the receptors for pertussis toxin (PT), one of the dominant virulence factors of *B. pertussis* [7], [9], [10]. LPS recognition by TLR4 on dendritic cells induces a proinflammatory response, including IL-12 which supports the development of Th1 cells [12]. In infected mice we and others have established that functional TLR4 is required for an early interleukin (IL)-1β, tumor necrosis factor (TNF)-α, and interferon (IFN)-γ response that may enhance bacterial clearance, and thus, despite the proinflammatory nature of these cytokines, may limit pathology [3], [8].

Signaling through TLR4 functions also in vaccine-induced immunity to *B. pertussis* [6], [13]. Whole cell pertussis vaccine (WCV), which contains abundant LPS, can induce the development of Th1- and Th17-cells in mice that mediate protective cellular immunity to *B. pertussis* [6], [13]. This response is abrogated in *Tlr4*-defective mice. In contrast, protection induced with acellular pertussis vaccine (ACV), which contains no or limited LPS, was compromised, but not completely abrogated in *Tlr4*-defective mice. In addition, we have observed that a lower PT-specific antibody response is associated with the minor allele of a single nucleotide polymorphism (SNP) (rs2770150) in *TLR4* in one-year-old WCV-vaccinated children [5]. Unfortunately the functional significance of this SNP is still unknown. This antibody response correlates with protection against disease both in humans [14]–[16] and in mice [17].

TLR4 interacts with adaptor molecules, interacting proteins, effectors, downstream pathways and target genes, which together constitute the TLR signaling pathway. This pathway functions as a complex, mutually coherent system of functionally interacting molecules. Genes in such a pathway may be regulated together. Indeed, array expression analysis of the *Tlr*-signaling pathway in *B. pertussis*-infected mice revealed that 16 of the 47 genes within the Toll-like receptor signaling pathway were regulated upon *B. pertussis* infection [4]. Thus, the investigation of genetic associations and gene-gene interactions in this pathway may provide novel insights into the role of the TLR signaling pathway, and especially of protein-protein interactions in this pathway, in the host response to infection and vaccination.

The role of the TLR signaling pathway, and genetic variation therein, in vaccine-induced immunity in humans has so far received little attention [18], although TLR-mediated activation of dendritic cells and B cells, induction of cytokine expression, and reversal of tolerance are crucial steps in the induction of immunity. Moreover, in addition to the microbial antigens in vaccines, several vaccine adjuvants have now clearly been identified as TLR ligands [19]. The current study therefore aims to provide further insight into the role of genes and gene-gene interactions in the TLR signaling pathway in the response to pertussis vaccination in humans.

## Materials and Methods

### Study cohort

We collected capillary blood samples from 855 one-year-old children from the KOALA Birth Cohort Study, the Netherlands. The design and procedures of the KOALA cohort study have been described previously [20]. Participants were asked to give written informed consent to sampling of their infant's buccal swab as source of DNA, and capillary blood at the age of 1 year for the determination of serologic parameters. All participants had signed the informed consent. Ethical approval was obtained from the medical ethics committee of the Maastricht University/University Hospital of Maastricht. Genetic testing was included in the medical ethical committee and informed consent procedures.

The children were vaccinated when they were 2, 3, 4, and 11 months old in the framework of the Dutch National Vaccination Program. We selected 704 children of which the parents indicated that they were vaccinated 4 times with WCV-containing diphteria-tetanus-pertussis-polio-*Haemophilus influenzae* type B vaccine. Parents were asked to collect buccal swabs for DNA from their child. We determined genotypes in 522 children. We excluded 5 children because their PT-IgG level was above 200 U/ml, which is indicative for a natural infection [21]. We also excluded 23 children because their mother, or both mother's parents were not born in the Netherlands. A further 4 children were excluded because less than 75% of their genotypes were available for analysis. Thus, data from 490 children were available for analysis.

### Antibody assay

The level of PT-specific immunoglobulin G (PT-IgG) was determined by enzyme-linked immunosorbent assay on capillary blood samples as described [21].

### SNP selection and genotyping

**Figure 1:**
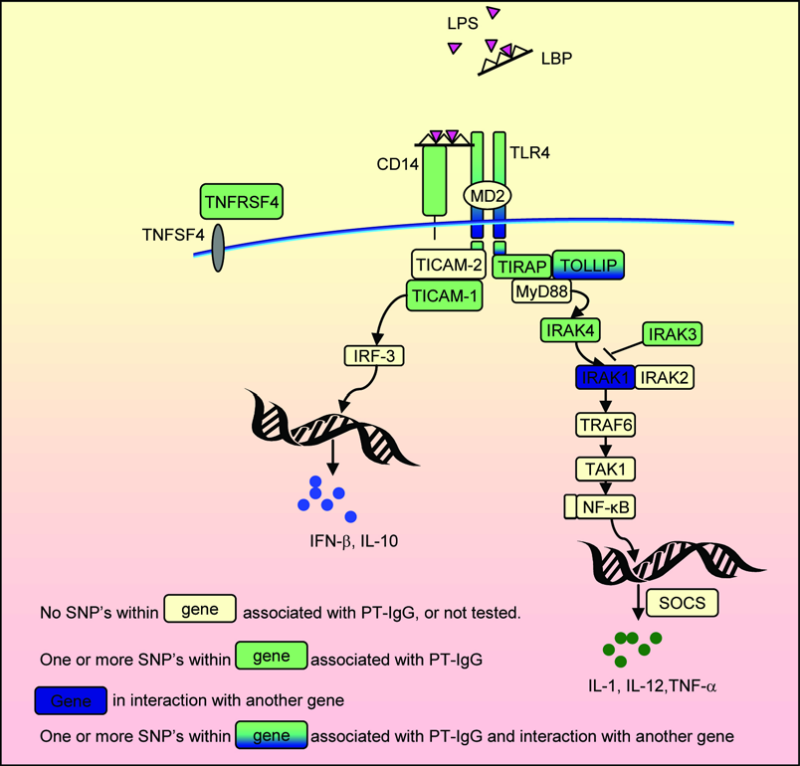
Summary of the TLR pathway in antigen-presenting cells and the main results of this paper. TLRs recognize molecular patterns associated with a broad range of pathogens including bacteria, fungi, protozoa and viruses. Vaccine components in WCV vaccine that may be recognized by TLR4 include LPS and PT. Following TLR4 activation, both the MyD88 and TICAM1 routes, leading to the expression of proinflammatory cytokines and type I IFNs respectively, may be activated. These promote the development of helper T cell responses providing T-cell help to B cells. TLR signaling in B cells may further promote the generation of antibody responses and the maintenance of serologic memory. TNFRSF4 is expressed on activated T cells. Adapted from BioCarta (http://www.biocarta.com/pathfiles/h_tollPathway.asp), KEGG (Kyoto Encyclopedia of Genes and Genomes) [50], and Metacore™ (http://www.genego.com/metacore.php).

Based on the recent literature and assisted by the online pathway-visualization program Metacore™ (http://www.genego.com/metacore.php) we have made a selection of genes in the TLR signaling pathway that could be associated with pertussis vaccine-induced antibody responses (Fig. 1). Haplotype tagging SNPs were selected from the HapMap database (http://www.hapmap.org/) or from the Innate Immunity web site (http://www.innateimmunity.net/datahomology) depending on the largest number of SNPs with a minor allele frequency >0.1 available in each database. Because pertussis vaccines may modulate the balance between Th1 and Th2 immunity [22], we further screened the biomedical literature until October 2005 for SNPs within the candidate genes known to have functional impact on, or to be associated with Th2 diseases, notably asthma and atopy. SNPs were named according to the Human Genome Variation Society guidelines (http://www.hgvs.org/mutnomen/recs.html). Rs numbers have been derived from the NCBI database (http://www.ncbi.nlm.nih.gov/sites/entrez).

Genomic DNA was extracted from buccal swabs or blood by chloroform-2-propanolol extraction [23]. DNA was amplified by using REPLI-g UltraFast technology (Qiagen™). Genotyping was performed by Competitive Allele-Specific PCR using KASPar™ genotyping chemistry, performed under contract by K-Biosciences (Cambridge, United Kingdom, http://www.kbioscience.co.uk). Quality of genotype data was verified as described previously [24]. Briefly, we verified the genotyping quality by three steps: 1) a number of samples were genotyped in both genomic and amplified DNA; 2) inheritance of alleles between parents and children was checked using FBAT (http://biostat.harvard.edu); 3) genotype data were analyzed for deviations from Hardy-Weinberg equilibrium using χ^2^ statistics. Comparison of genotypes between genomic and amplified DNA and evaluation of inheritance patterns between parents and children revealed an excellent quality of the genotypes with a genotyping error of <1%. Table 1 summarizes the genes and number of SNPs analyzed.

**Table 1:** Genes and number of SNPs analyzed for association with vaccine-induced PT-specific immunoglobulin B response.

Gene	Number of haplotype-tagging SNPs	Description
*LBP*	8	LPS-binding protein
*CD14*	5	Monocyte differentiation antigen CD14
*TLR4*	8	Toll-like receptor 4
*TOLLIP*	14	Toll-interacting protein
*TIRAP*	5	TIR domain-containing adaptor protein
*TICAM1*	2	TIR domain-containing adaptor molecule 1
*TICAM2*	4	TIR domain-containing adaptor molecule 2
*TRAF6*	2	TNF receptor-associated factor 6
*MYD88*	2	Myeloid differentiation primary response gene 88
*IRAK1*	3	Interleukin 1 receptor-associated kinase 1
*IRAK3*	5	Interleukin 1 receptor-associated kinase 3
*IRAK4*	2	Interleukin 1 receptor-associated kinase 4
*SOCS1*	2	Suppressor of cytokine signaling 1
*TNFRSF4*	4	TNF receptor superfamily, member 4
*TNFRSF14*	2	TNF receptor superfamily, member 14
*TNFRSF18*	1	TNF receptor superfamily, member 18
*TNFSF4*	6	TNF ligand superfamily, member 4

Genes are named according to the nomenclature of the Human Genome Organisation (HUGO) (http://www.hugo-international.org).

SNP: single nucleotide polymorphism.

### Statistical analyses

We used three parameters to examine genetic associations. First, we compared cases and controls defined as children with PT-IgG titers in the most extreme 10^th^ percentiles. Secondly, we performed analyses with cases and controls defined as children with PT-IgG titers in the most extreme 33^rd^ percentiles. The 10^th^ and 33^rd^ percentiles reflect the values of the PT-IgG titers below or above which 10 or 33 percent of the observations were found. The highest and lowest 10^th^ percentiles (54 and 49 children respectively) were chosen because we previously found a SNP in TLR4 (rs2770150) associated with the lowest 10^th^ percentile (low responders) compared to the highest 10^th^ percentile (high responders) titer of PT-IgG (*p* = 0.027) [5]. The highest and lowest 33^rd^ percentiles (154 and 166 children respectively) were chosen to obtain sufficient cases in the Multifactor Dimensionality Reduction (MDR) analyses. In addition we examined genetic association with PT-IgG titers in a continuous analysis, because IgG titers represent a continuous variable therewith circumventing arbitrary cut-off values.

Data from 4 boys were excluded in the MDR analyses since they had a heterozygous genotype for an IRAK1 SNP located on the X-chromosome. Thus for the MDR analyses data from 153 and 164 children were available with PT-IgG in the lowest and highest 33^rd^ percentiles, respectively.

After 10 based logarithmic transformation, the PT-IgG levels were normally distributed according to Levene's test (*P*>0.05). To examine possible confounding factors, we tested for associations between PT-IgG titer and the number of days between vaccination and blood sampling and infant gender using Pearson correlation. None of the factors tested influenced the PT-IgG level (*P*>0.05).

We examined whether SNPs were in Hardy Weinberg equilibrium (HWE) using the chi-square test. As a result we excluded the following SNPs because they showed a significant deviation (*p*<0.001) from HWE: *IRAK4* (rs146156), *TOLLIP* (rs5743854), and *TLR4* (rs10759931). Two SNPs in *SIGIRR* were excluded because genotyping failed to give consistent results. Therewith 75 SNPs were available for analyses. As indicated above genotype data on *IRAK1* SNPs (located on the X-chromosome) were excluded from 4 boys who had a heterozygous genotype.

Univariate associations between genotypes and log PT-IgG titers were assessed by analysis of variance (ANOVA). When associations reached *p*-values<0.10, we further determined the best fitting genetic model (co-dominant, dominant, recessive or additive) in linear regression analysis. In addition we compared the distribution of alleles and genotypes among individuals with IgG in the highest 10^th^ or 33^rd^ percentiles with that of individuals with IgG in the lowest 10^th^ or 33^rd^ percentiles using Pearson's chi-square test. To control for multiple testing, we calculated the false discovery rate (FDR) according to Benjamini and Hochberg [25].

Gene-gene interactions were studied using Multifactor Dimensionality Reduction (MDR version 1.0.0). MDR has been described in detail by Ritchie et al [26]. Briefly, this method reduces dimensionality of multifactor information to one dimension, i.e. high risk or low risk. First, the data are divided into a training set (9/10 of the data) and an independent testing set (1/10 of the data). The model with the best classification error is selected from the training set and the prediction error of that model is estimated using the testing set. This procedure is repeated 10 times, and the model with the combination of loci that maximizes the cross-validation consistency and minimizes the prediction error is selected. Cross-validation consistency is a measure of the number of times an MDR model is identified in each possible group of nine-tenths of the subjects. To obtain sufficient cases we performed MDR for individuals with IgG in the highest 33^rd^ percentile defined as cases, and individuals with IgG in the lowest 33^rd^ percentile as controls. For MDR we excluded 5 SNPs (rs2569190, rs5743894, rs5743859, rs5743987, and rs4986791) that were in strong linkage disequilibrium (LD) with other SNPs using a cut-off value of r^2^>0.80. LD was calculated using the software program Haploview. Because missing data are not allowed in MDR, missing SNPs were imputed using multiple imputation (MICE) [27]. MDR analyses were done separately using 5 imputation files, and subsequently average testing accuracies and cross-validation consistencies were calculated. The model with the highest average cross validation consistency and lowest average prediction error was selected as “best model”. Average cross validation consistency is the number of times the model was selected as the best model after 10-fold cross-validation runs. Average testing balanced accuracy is the accuracy of classification of cases and controls in the testing dataset (one-tenth of the data) calculated as (Sensitivity+Specificity)/2. To examine the significance of testing accuracies, we determined 1,000 MDR analyses for each test after permutation (200 of each imputation file). The null-hypothesis was rejected when the one-sided *p*-value, as estimated by 1,000 permutations in Monte Carlo simulation, was <5%. In that case the “best model” predicts the status of cases and controls better than chance without the need to correct for multiple testing. Interactions revealed by MDR analysis were confirmed by linear regression (continuous data) and by logistic regression (33^rd^ percentiles). Finally, interactions were visualized by constructing an interaction dendrogram according to Moore et al [28]. This dendrogram visualizes how much information about case-control status is gained by combining two or more SNPs using the MDR based on measures of entropy.

## Results

### Single gene associations

In total 9 haplotype-tagging SNPs in 8 genes were significantly associated with PT-IgG levels (table 2). *P*-values were first calculated without prior assumption of the mode of inheritance (2 df). When *p* reached a value<0.10, the *p*-value belonging to the best fitting genetic model was subsequently calculated. Table 3 gives odds ratios (ORs) belonging to the best fitting genetic model for individuals in the highest 10^th^ or 33^rd^ percentile compared to individuals in the lowest 10^th^ or 33^rd^ percentile, and table 4 shows the PT-IgG titers of individuals with the indicated genotypes. The results confirmed the association of a SNP (rs2770150) in TLR4 as described by Banus et al [5], but only in the test that compares the extreme 10^th^ percentiles. In addition we found associations between PT-IgG titer with SNPs in CD14, TOLLIP, TIRAP, IRAK3, IRAK4, TICAM1, and TNFRSF4. In the genetic models the ORs for SNPs that showed association in one or more tests was between 1.73 and 3.56 for alleles with a positive effect, and between 0.28 and 0.45 for alleles with a negative effect (table 3), indicating that individual genotypes appeared to have small to moderately strong main effects. The three tests (continuous analysis, 10^th^ and 33^rd^ percentiles) appeared to complement each other, with the least number of associations found in the tests comparing the highest and lowest 33^rd^ percentiles. Two TOLLIP SNPs (rs5744034 and rs5743894) that were almost completely in linkage disequilibrium (D' = 1; r^2^ = 0.986), gave statistically significant associations in all three tests with the lowest *p*-values, and displayed a dominant mode of inheritance. None of the SNPs was significantly associated with the PT-IgG titer after controlling for multiple testing by FDR analysis.

**Table 2:** Single-nucleotide polymorphisms (SNPs) in genes from the TLR signaling pathway significantly associated with vaccine-induced PT-immunoglobulin G.

Gene	Rs-number	Alleles^a^	MAF^b^	continuous analysis *p*-value^d^	10^th^ percentiles^c^ *p*-value^d^	33^rd^ percentiles^c^ *p*-value^d^
*CD14*	rs5744455	T/C	0.24	**0.03**	0.12	**0.02**
*TLR4*	rs2770150	C/T	0.27	0.11	**0.04**	0.18
*TOLLIP*	rs5744034	C/T	0.18	**0.04**	**0.03**	**0.05**
*TOLLIP*	rs5743894	G/A	0.17	**0.04**	**0.04**	**0.08**
*TIRAP*	rs8177376	G/T	0.24	**0.08**	**0.05**	0.31
*TICAM1*	rs1046673	T/C	0.15	**0.06**	0.07	0.12
*IRAK3*	rs3782347	G/A	0.28	0.51	**0.08**	0.61
*IRAK4*	rs4251520	C/T	0.12	**0.07**	**0.02**	0.28
*TNFRSF4*	rs17568	A/G	0.20	**0.07**	0.22	0.14

**Bold p-values become <0.05 for the best fitting genetic model**.

^a^ Minor allele first; ^b^ MAF = minor allele frequency; ^c^ highest versus lowest; ^d^ *P*-values without prior assumption of the mode of inheritance (2 df).

**Table 3:** Odds ratios of significantly associated genotypes with vaccine-induced PT-immunoglobulin G.

Gene	Rs-number	10^th^ percentiles			33^rd^ percentiles		
		Best fitting genetic model	Odds ratio^a^ (95% CI^b^)	*p*-value	Best fitting genetic model	Odds ratio^a^ (95% CI^b^)	*p*-value
*CD14*	rs5744455			*NS*	recessive (TT vs. TC/CC)	0.28 (0.11–0.73)	0.009
*TLR4*	rs2770150			*NS*			*NS*
*TOLLIP*	rs5744034	dominant (CT/CC vs. TT)	2.93 (1.16–7.40)	0.02	dominant (CT/CC vs. TT)	1.79 (1.11–2.90)	0.02
*TOLLIP*	rs5743894	dominant (GA/GG vs. AA)	2.68 (1.06–6.79)	0.04	dominant (GA/GG vs. AA)	1.73 (1.07–2.79)	0.03
*TIRAP*	rs8177376	Additive^c^ (number of G alleles)	0.45 (0.23–0.87)	0.02			*NS*
*TICAM1*	rs1046673			*NS*			*NS*
*IRAK3*	rs3782347	Additive^c^ (number of G alleles)	1.94 (1.01–3.73)	0.05			*NS*
*IRAK4*	rs4251520	dominant (CT/CC vs. TT)	3.56 (1.38–9.18)	0.009			*NS*
*TNFRSF4*	rs17568			*NS*			*NS*

a OR belonging to the best fitting genetic model.

b Confidence interval.

c On additive scale in a logistic regression model, i.e. multiplicative.

**Table 4:** Influence of single-nucleotide polymorphisms (SNPs) on vaccine-induced PT-immunoglobulin G titers.

Gene	Rs-number	Genotype	Number	PT-IgG titer Geometric Mean (95% CI)	Best Model	Ratio IgG titers (95% CI)	*p*-value
*CD14*	rs5744455	CC	276	27.1 (25.2–29.0)			
		CT	168	25.8 (23.8–28.0)	Recessive	0.76 (0.61–0.94)	0.01
		TT	30	20.2 (16.7–24.3)			
*TLR4*	rs2770150	TT	255	25.7 (24.0–27.6)			
		CT	198	27.3 (25.2–29.7)			0.11
		CC	34	22.0 (18.5–26.3)			
*TOLLIP*	rs5744034	TT	321	24.9 (23.3–26.5)			
		CT	149	28.7 (26.3–31.4)	Dominant	1.15 (1.03–1.28)	0.01
		CC	11	26.6 (19.7–36.0)			
*TOLLIP*	rs5743894	AA	326	24.9 (23.3–26.5)			
		GA	146	28.7 (26.3–31.4)	Dominant	1.14 (1.02–1.27)	0.02
		GG	9	23.7 (17.3–32.5)			
*TIRAP*	rs8177376	TT	284	36.3 (29.5–44.4)			
		TG	168	28.4 (21.8–36.9)	Additive	0.91 (0.84–0.99)	0.03
		GG	32	18.7 (9.6–34.9)			
*TICAM1*	rs1046673	CC	355	34.8 (29.0–41.7)			
		CT	117	27.3 (19.6–37.6)	Additive	0.90 (0.81–0.99)	0.03
		TT	12	11.4 (3.13–35.9)			
*IRAK3*	rs3782347	AA	248	30.0 (23.8–37.7)			
		GA	202	34.0 (26.9–42.7)			0.51
		GG	34	41.8 (22.7–74.2)			
*IRAK4*	rs4251520	TT	372	29.5 (24.6–35.2)			
		CT	104	43.5 (31.0–60.3)	Additive	1.14 (1.02–1.27)	0.02
		CC	8	66.0 (8.3–366.8)			
*TNFRSF4*	Rs17568	GG	302	33.9 (27.9–41.0)			
		GA	151	37.0 (27.3–49.6)	Recessive	0.75 (0.58–0.96)	0.02
		AA	21	13.8 (5.9–30.1)			

### Gene-gene interactions

**Figure 2:**
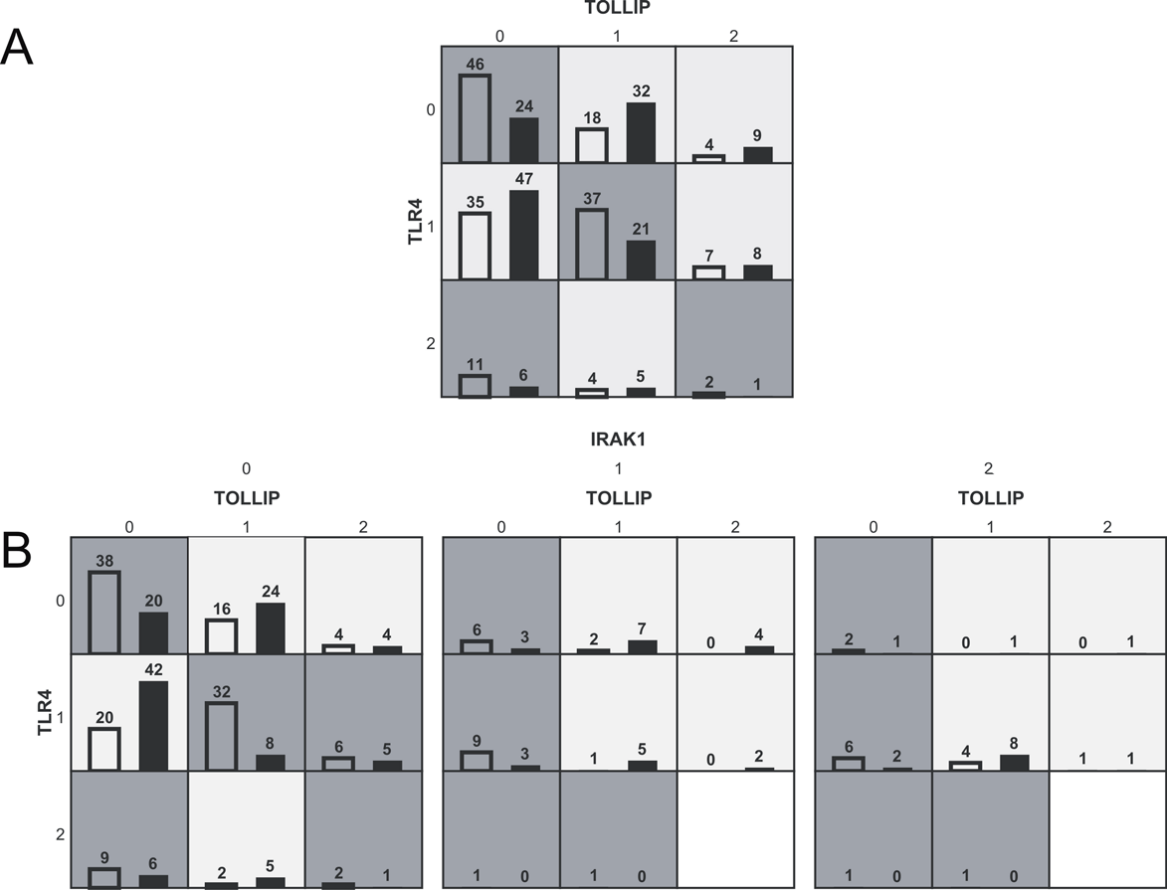
Genetic interactions between *TOLLIP*, *TLR4*, and *IRAK1*. Graphical display of evidence observed in MDR analysis of interaction among SNPs in TOLLIP (rs4963060), TLR4 (rs6478317) and IRAK1 (rs1059703). For each cell the left bar (empty) represents the number of children with PT-IgG in the highest 33^rd^ percentile (total: 164) and the right bar (dark) the number of children with PT-IgG in the lowest 33^rd^ percentile (total: 153) for the specific combination of 2 (fig 2A) or 3 genotypes (fig 2B). When the ratio is >164/153 the cell indicates a higher chance on a titer in the highest 33^rd^ percentile (dark background), and when the ratio is <164/153 the cell indicates a higher chance on a titer in the lowest 33^rd^ percentile (light background). 0, 1, and 2 represent the number of minor alleles.

MDR analyses revealed two significant genetic interactions of SNPs in *TOLLIP* (rs4963060), *TLR4* (rs6478317; syn.: rs2737190) and *IRAK1* (rs1059703) with cases in the highest 33^rd^ percentile compared to controls in the lowest 33^rd^ percentile of PT-IgG titers (Table 5, Fig. 2a and Fig. 2b).

**Table 5:** Results of gene-gene interactions analyzed by MDR.

Genes and rs numbers	Average testing balanced accuracy^a^ (%)	Average cross validation consistency^b^	*P* c
*TOLLIP* (rs5744034)	57	7.4	0.09
*TOLLIP* (rs4963060)*TLR4 (rs6478317)	61	6.4	0.01
*TOLLIP* (rs4963060)*TLR4 (rs6478317)*IRAK1 (rs1059703)	64	8.2	0.01

a Average testing balanced accuracy is the accuracy of classification of cases and controls in the testing dataset (one-tenth of the data) calculated as (Sensitivity+Specificity)/2.

b Average cross validation consistency is the number of times the model was selected as the best model after 10-fold cross-validation runs.

c Significance of accuracy (empirical *p*-value based on 1000 permutations).

**Figure 3:**
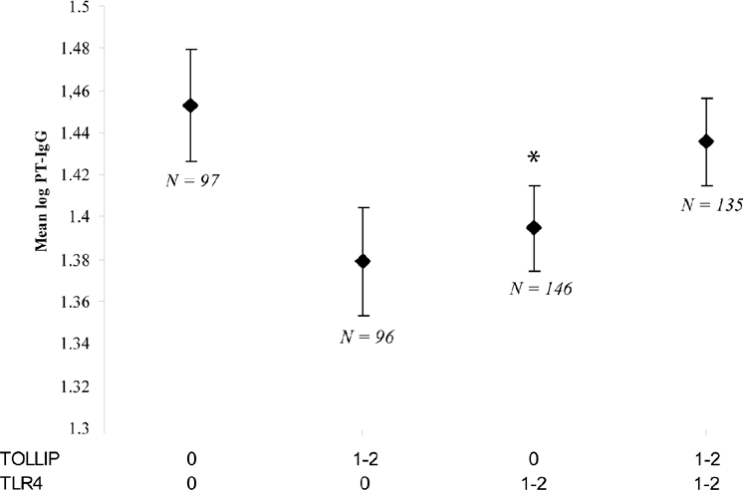
Influence of *TOLLIP* and *TRL4* and their interaction on log PT-IgG titers. Influence of *TOLLIP* (rs4963060) and *TLR4* (rs6478317) interaction on log PT-IgG titers following pertussis vaccination in a dominant model of inheritance. The presence of one or two minor alleles of one of these SNPs, but not the presence of one or two minor alleles of both SNPs, was associated with a lower PT-IgG titer. 0, 1, and 2 represent the number of minor alleles. * Significantly different from the group homozygous for the wild-type alleles (*p* = 0.04). *P* interaction term = 0.01. Vertical bars indicate standard errors.

First, a combination of a *TOLLIP* SNP (rs4963060) and a *TLR4* SNP (rs6478317) had a strong synergistic effect on PT-IgG titers (*p* = 0.01; average testing balanced accuracy 61%). The interaction was confirmed by linear regression analysis for the level of PT-IgG as the outcome (*p*-value for interaction term = 0.01), and by logistic regression analysis for the highest 33^rd^ percentile compared to the lowest 33^rd^ percentile (*p*-value for interaction term = 0.0005). Figure 2 shows which combinations of genotypes have relatively high levels of PT-IgG, i.e. dark shaded cells with a high ratio of cases (left bars) and controls (right bars). Especially children with wild type genotypes of both TOLLIP (rs4963060) and TLR4 (rs6478317) had a high chance of PT-IgG in the highest 33^rd^ percentile (Fig. 2A, Fig. 3). The strong and significant interaction between the TOLLIP (rs4963060) and TLR4 (rs6478317) SNPs was further indicated by a positive information gain based on measures of entropy [28], [29]. That is, information about case-control status is gained above that provided by the SNPs individually by combining the two SNPs (data not shown).

Second, an IRAK1 SNP (rs1059703) when added to the same combination (TOLLIP rs4963060 and TLR4 rs6478317) further increased the accuracy (table 5), and this combination was also significantly associated with PT-IgG titers (*p* = 0.01). However, Fig. 2B shows that most of the interactive effect must be attributed to TOLLIP and TLR4. Of note is that the SNPs showing genetic interaction were not associated with the vaccine responses in univariate analyses.

## Discussion

Following earlier studies that provided evidence for the involvement of TLR4 in vaccine-induced immunity to *B. pertussis*, we elaborated on the role of genetic differences in other genes in the TLR signalling-pathway. This approach was based on the following. First, that TLR4 would likely function in a network of molecules, together forming a molecular pathway. Second, exploiting genetic differences in vaccine-induced responses in a cohort of vaccinated children would constitute a feasible approach to point to the functional role of genes that may be interesting to study in follow-up studies. Third, genes that play a role in *B. pertussis* vaccine-induced immunity may also be important in the response to other vaccines or the pathobiology of infection. Indeed, so far there is evidence that TLR4 affects both vaccine responses, as well as the course of infection with gram-negative bacteria and certain viruses, including respiratory syncytial virus [3], [5], [6], [30], [31].

This study indeed indicated association of haplotype-tagging SNPs in several genes in the TLR signaling pathway (*CD14*, *TLR4*, *TOLLIP*, *TIRAP*, *IRAK3*, *IRAK4*, *TICAM1* and *TNFRSF4*) with the PT-specific antibody response following vaccination. This is an interesting observation that needs nevertheless to be interpreted with caution. Association between a gene polymorphism and a phenotype in a genetic-epidemiological study may, in isolation, convey little information about the causative role of the polymorphism. Besides identifying a real causative polymorphism directly responsible for the phenotype, allelic association may point to a polymorphism that is in close linkage disequilibrium to the associated marker. Indeed, as the haplotype-tagging SNPs are markers which capture most of the haplotypes in a region of linkage disequilibrium, and most associated SNPs have an unknown or no biologic function (summarized in Table 6), it is likely that the observed associations refer to genetic haplotype variation tagged by these SNPs. Such linkage disequilibrium, as well as gene-environment interactions, might vary between populations, and as a result association studies may yield conflicting results [32]. In addition, multiple testing in association studies may result in false discoveries, which necessitate the development of strategies to underline the evidence of gene involvement in a phenotype [33]–[35]. The single SNP associations found in this study did not withstand correction for multiple testing by FDR analysis (data not shown). Additional data are therefore needed to corroborate our findings. These may be derived by replication in other genetic-epidemiological studies, preferably in combination with studies on the functional role of gene products that provide biological plausibility. For example, it would be interesting to know whether individuals genetically different in TLR-pathway genes have altered proinflammatory cytokine production upon vaccination. These data are unavailable for the children from our cohort. However, in our murine model of wP vaccination and *B. pertussis* challenge, Tlr4 enhanced the production of the proinflammatory cytokines IL-1α, IL-1β, and TNF-α [13].

**Table 6:** Summary of locations and functional data of SNPs in genes from the TLR signaling pathway significantly associated with vaccine-induced PT-immunoglobulin G.

Gene	Rs-number	Alleles^a^	Location	Possible functional significance
*CD14*	rs5744455	T/C	−651	promoter
*TLR4*	rs2770150	C/T	−3612	promoter
*TLR4* b	rs6478317	G/A	−2570	promoter
*TOLLIP*	rs5744034	C/T	34491, exon 6	3′ UTR
*TOLLIP*	rs5743894	G/A	5956, intron 1	intron
*TOLLIP* b	rs4963060	G/A	9668, intron 1	intron
*TIRAP*	rs8177376	G/T	2822, exon 6	3′ UTR
*TICAM1*	rs1046673	T/C	2148, 3′ near gene	3′ UTR
*IRAK1* b	rs1059703	T/C	12–36, intron 11; 6434, coding exon	intron boundary; amino acid change 453 S/L
*IRAK3*	rs3782347	G/A	48836, intron 8	intron
*IRAK4*	rs4251520	C/T	13423, intron 8	intron
*TNFRSF4*	rs17568	A/G	2085, exon 5, amino acid position 178	synonymous mutation

^a^ Minor allele first; ^b^ associated in interaction.

Data are based on the NCBI database (http://www.ncbi.nlm.nih.gov/sites/entrez) and the Innate Immunity web site (http://www.innateimmunity.net).

The multiple testing problem does not apply to the strong genetic interaction we found between SNPs in *TLR4*, *TOLLIP*, and *IRAK1*. MDR chooses one best model from all possible combinations of attributes. Subsequently we tested the estimated accuracy of the best model for significance using the distribution of accuracies of best models under the null-hypothesis obtained after 1000 permutations. Therefore, the problem of multiple significance testing that inflates the type 1 error rate in single SNP analyses does not apply to MDR analyses. These genetic interactions therefore provide strong evidence that these genes are indeed involved in the response to vaccination and work together as functionally related genes in a single biological module.

We employed three different analyses, i.e. a continuous analysis and analyses comparing study subjects with the extreme 10^th^ and 33^rd^ percentiles in PT-IgG titer. Though not independent, these different analyses appeared to be complementary and confirmatory. The evidence of association was most consistent and strong for the SNPs in the *TOLLIP* gene, which showed association in all three analyses (at least at the genotype level) and also had the lowest *p*-values (Table 2). A role of TOLLIP in vaccine-induced immunity also appears plausible from its biological role and interactions in the TLR signaling pathway. TOLLIP is a small protein that binds the activated IL-1 receptor type I (IL-1RI) complex, as well as TLR2 and TLR4 complexes [36], [37]. TOLLIP also suppresses IRAK-1's kinase activity. In mice TOLLIP deficiency results in attenuated responses of the proinflammatory cytokines, IL-6, and TNF-α upon stimulation with IL-1ß and low doses of LPS. TOLLIP therefore likely acts in “fine tuning” or coordinating optimal signaling through IL-1RI and TLR4 [38]. We consider that physical interaction between TLR4, TOLLIP and IRAK1 or their concerted regulation may explain the genetic interaction of these genes. Although further work should proof that the SNPs (or SNPs in linkage disequilibrium) affect the physical interaction between these proteins, the agreement between physical and genetic interaction between functionally relevant genes within a single biological pathway is, to our knowledge, a unique finding.

In addition to its interaction with TOLLIP, TLR4 together with CD14 and the myeloid differentiation protein-2 (MD-2), forms a pattern recognition receptor that plays an initiating role in the innate immune response to LPS from Gram-negative bacteria. The current view is that CD14 conveys LPS to the TLR4/MD-2 complex [39]. Evidently this cooperative function may explain the genetic association of CD14 with the WCV vaccine response found in this study. Previously genetic variation in another promoter SNP in *CD14* has been shown to affect pneumococcal vaccine responsiveness in children. The T allele of the *CD14* C-159T polymorphism (rs2569190) was associated with increased serum CD14 levels, and TT homozygotes showed higher serotype-specific anti-pneumococcal vaccine IgG antibody levels [40].

The associations of *TIRAP* SNP rs8177376 and *TICAM1* SNP rs1046673 may also be explained by physical interaction or concerted regulation between TIRAP and TICAM1 with TOLLIP and TLR4. TIRAP and TICAM1 belong to the Toll/Interleukin-1 receptor (TIR) domain-containing adaptors, also including MyD88, that modulate TLR signaling pathways. TLRs thus can activate two distinct branches of downstream signaling pathways [41]. Our data suggest that both pathways are induced following WCV pertussis vaccination.

The interleukin-1 receptor-associated-kinases (IRAKs) are signal transduction mediators of the Toll and IL-1 receptor (IL-1R) families. IRAK3 and IRAK4 showed evidence for association in this study. These proteins may be involved in vaccine-induced immunity because they regulate TLR signaling and innate immune homeostasis [42].

Finally TNFRSF4 (syn. OX40 antigen, ACT35 antigen, CD134) showed evidence for association. TNFRSF4 is a cell surface antigen on T lymphocytes and a member of the tumor necrosis factor/nerve growth factor receptor family [42], [43]. Ligation of TNFRSF4 during T-cell-dendritic cell interaction is crucial for clonal expansion of antigen-specific T-cells and generation of T-cell memory [45]. Munks et al [46] found in mice that stimulation of TNFRSF4 increased the number of antigen-specific CD4 T cells following vaccination. Therefore it was suggested that stimulants of TNFRSF4 (and other members of its family) can improve the response to vaccination and may be useful as vaccine adjuvants [46], [47].

Together, the majority of our findings point to the significance of directly or indirectly physically interacting proteins at the extra- and intracytoplasmic sites of the cell membrane of antigen presenting cells, or B cells, or both, in vaccine responsiveness. In particular we have identified significant interactions between *TOLLIP*, *TLR4*, and *IRAK1*. Further studies are required to explain how and where the genetic associations and interactions in the TLR pathway affect the PT antibody response. A T-dependent antibody response is clearly a multi-step process, and the TLR signalling pathway is likely involved in several of these steps. TLR signalling induces dendritic cell maturation and helper T cell activation, which are required to provide T-cell help to B cells. This may occur directly, or indirectly via cytokines, including IL-12 and TNF-α. In addition, activation of the TLR signaling pathway in B cells may be helpful or required for the generation of T-dependent antibody responses. This may occur by promoting antigen presentation by antigen-specific B cells and by promoting maturation of B cells into antibody-producing plasma cells [48]. In addition, TLR signaling in memory B cells may contribute to the maintenance of serological memory [49]. Altogether, we conclude that our combined data are a strong indication for the involvement of the TLR signaling pathway in the response to vaccination, as well as for the cooperation of its genes in a functional interacting network.
